# Safety and Efficacy of Long-Term Zoledronic Acid in Advanced Breast Cancer with Bone Metastasis in South China

**DOI:** 10.1155/2020/5670601

**Published:** 2020-09-30

**Authors:** Qianyu Wang, Guifang Guo, Zhaohui Ruan, Huijiao Cao, Ying Guo, Long Bai, Chang Jiang, Shousheng Liu, Wenzhuo He, Jinsheng Huang, Yuming Rong, Xuxian Chen, Liangping Xia, Bei Zhang, Roujun Peng

**Affiliations:** ^1^Department of VIP Section, Sun Yat-sen University Cancer Center, State Key Laboratory Oncology in South China, Collaborative Innovation Center of Cancer Medicine, Guangzhou 510060, China; ^2^Department of Clinical Research, Sun Yat-sen University Cancer Center, State Key Laboratory Oncology in South China, Collaborative Innovation Center of Cancer Medicine, Guangzhou 510060, China

## Abstract

**Background:**

This retrospective study aimed to characterize the long-term (>24 months) safety profile of zoledronic acid (ZA). We aimed to investigate whether long-term ZA treatment had greater benefits than short-term treatment in patients from southern China with advanced breast cancer (ABC) with bone metastasis. *Patients and Methods*. A total of 566 metastatic breast cancer cases were included and divided into two groups according to the duration of ZA treatment. The included patients had at least one lytic bone lesion and had no skeletal-related events (SREs) prior to ZA therapy. The primary endpoint was to analyze the safety and long-term adverse effects, which covered osteonecrosis of jaws (ONJ), renal impairment, and hearing impairment. The second objective was to determine the efficacy of long-term ZA treatment by the incidence of SREs.

**Results:**

Fifteen patients were diagnosed with ONJ (2.7%): nine in the short-term group (3.1%) and six in the long-term group (2.2%, *P* = 0.606). Five cases (0.9%) had renal function impairment: two in the short-term group (0.7%) and four in the long-term group (1.1%, *P* = 0.676). One patient (0.2%) in the long-term group had hearing impairment after 23 months of ZA treatment (0.4%, *P* = 0.482). In total, 103 cases in the short-term group (35.2%) and 138 cases in long-term group (50.5%) developed SREs (*P* < 0.001). The mean annual SRE rate was 0.3 in the short-term group (range, 0–3.1) versus 0.2 in the long-term group (0–1.0, *P* = 0.269). Subgroup analysis suggested that cases with non-load-bearing bone involvement and those who received systematic anticancer therapy without chemotherapy might benefit from long-term ZA treatment. Cox regression analysis indicated poor performance status, and nonvisceral organ involvement predicted high risk for SRE.

**Conclusions:**

The extension of ZA treatment did not increase the long-term adverse events and reduced the annual incidence of SREs beyond 24 months. Although longer treatment of ZA over 24 months appeared to be safe, further prospective investigation is required.

## 1. Introduction

Bone is the most frequent metastatic site in advanced breast cancer (ABC), accounting for approximately 65% to 75% of metastatic cases [1]. Bone lesions change their metabolism and mobility [2], which increases the risk of skeletal complications and has a detrimental impact on patients' quality of life [1].

Zoledronic acid (ZA) is a third-generation heterocyclic nitrogen-containing bisphosphonate (BP) that has been demonstrated to have higher efficacy than other BPs in clinical trials [3–5]. ZA is effective in minimizing bone loss and delaying the onset and reducing the risk of skeletal-related events (SREs) [3–8]. The American Society of Clinical Oncology (ASCO) guideline recommends the use of ZA for breast cancer patients with lytic bone metastasis [9].

Bone metastasis is incurable, and the risk of SRE increases as lifetime dilation [10]. It is currently unknown how long metastatic bone patients are treated with ZA, although guidelines suggest that patients should continue intravenous bisphosphonates until a substantial decline in their performance status is apparent [9, 11]. However, adverse events (AEs) are very common after the administration of ZA and include acute phase reactions (flu-like symptoms: low-grade fever, myalgia, bone pain, and headache), hypocalcemia, and hypophosphatemia, as well as long-term side effects, such as renal function impairment, hearing impairment, and osteonecrosis of jaws (ONJ) [12]. Chronic use of ZA has been associated with ONJ and atypical hip fractures [3–5]. It is important to determine whether long-term ZA treatment is as safe as short-term treatment or, indeed, whether long-term treatment confers any clinical benefits. Experts recommend that bisphosphonate use beyond 2 years should be personalized based on risk evaluation [9, 13]. ZA has been administrated up to 2 years [13, 14] or even longer in some cases [6, 7, 15, 16]. Furthermore, it has been reported that prolonged administration of bisphosphonates (pamidronate, ibandronate, or ZA) was similarly effective in northern Chinese ABC patients with bone metastasis, although there is currently no standard duration [9, 17]. The objective of this study was to explore the safety and efficacy of long-term (>24 months) compared to short-term (≤24 months) ZA treatment for breast cancer patients from southern China with bone metastasis.

## 2. Patients and Methods

### 2.1. Patients

Patients were recruited from Sun Yat-sen University Cancer Center from January 2005 to Oct 2018. The inclusion criteria were patients who were pathologically diagnosed with breast cancer and had at least one lytic metastatic bone lesion detected by imaging scan. All eligible cases received more than 6 months of ZA treatment without a SRE before ZA initiation and had complete clinical information and survival data. The exclusion criteria were as follows: (1) patients with other malignant carcinomas besides breast cancer; (2) patients who received any type of bone-modifying agent (BMA) other than ZA or ZA combined with another BMA; and (3) cases with incomplete clinical information or survival data. Anticancer therapy was permitted for all patients, and the therapeutic decisions were made by the physician.

The Institutional Ethics Committee at the Sun Yat-sen University Cancer Center approved the study.

### 2.2. Study Design and Treatment

This was a retrospective, self-controlled study, consisting of two parts of analysis. Patients were divided into two groups based on the period of ZA administration: group A (ZA 6–24 months) and group B (ZA > 24 months).

The first component of the study was safety analysis. We investigated the long-term adverse events (AEs) in patients treated with ZA; these AEs included ONJ, renal impairment, and hearing impairment, which are the most common long-term AEs in clinical studies and had clear diagnostic indicators. The safety analysis aimed to compare the cumulative incidence risk of AEs between the short- and long-term ZA groups.

ONJ was defined as an area of exposed or necrotic bone in patients who had received treatment with ZA without maxillary radiotherapy that failed to heal within 8 weeks. Renal impairment was defined as serum creatinine levels that increased by ≥0.5 mg/dL or 1.0 mg/dL from baseline for patients with baseline serum creatinine <1.4 mg/dL or >1.4 mg/dL, respectively, or serum creatinine that increased to at least twice that of the baseline value. Hearing impairment could be defined through pure tone audiometry (any cutoff eligible) or self-report.

The second component of the study was efficacy analysis, which aimed to compare the annual incidence of SREs, by comparing the time to first SRE between the two groups.

The final objective of the study was to determine cases who could benefit from long-term ZA treatment and explore the prognostic factors that predict the risk of SREs, defined as pathologic fractures, spinal cord compression, or surgery or radiotherapy to bone [11]. A new vertebral compression fracture was diagnosed by a decrease of ≥25% of the total, anterior, or posterior vertebral height from baseline [11].

Patients received an infusion of ZA 4 mg over 15 minutes, every 3–4 weeks. In some cases, the medication interval was changed to every 2–3 months beyond 2 years, depending on the clinicians' choice. Patients had regular dental examinations and accepted hormone therapy, chemotherapy, anti-HER2 target therapy, radiotherapy, or surgery according to the clinical protocol.

Routine blood tests, electrolyte analysis, and renal function and calcium tests were performed regularly once every 3 to 6 months in all cases.

### 2.3. Study Endpoints

The first endpoint was the long-term safety of ZA, as determined by evaluation of ONJ, renal impairment, and hearing impairment. All three AEs were collected from the initiation of ZA treatment to death or the last follow-up, in order to compare the cumulative incidence risk of AEs between the short- and long-term groups. The second endpoint was to compare the annual incidence of SREs between the two groups. Considering the imbalance of survival time or follow-up period, and the cumulative effect of SREs once bone lesion has occurred, the annual incidence of SREs was applied (number of SREs divided by the study time in years). For the annual incidence of SREs and multiple-event analyses, a 21-day event window was applied for counting SREs. Any event occurring within 21 days of a previous event was not included in the calculations, and the 21-day interval was not calculated as time at risk [18]. The third endpoint was to explore the onset of the first SRE after ZA treatment, the time of which was defined from the initiation of ZA to the first SRE diagnosis. We collected all SREs from the initiation of ZA to death or the last follow-up. The fourth endpoint was to distinguish who could benefit from long-term ZA treatment. The last endpoint was to investigate the prognostic factors that predicted the risk of SREs.

### 2.4. Statistical Analysis

Demographic and disease parameters were assessed between the two groups using Pearson *χ*^2^ test or Fisher's exact test for categorical factors, or Wilcoxon test for continuous factors. Cumulative survival probabilities were calculated through Kaplan–Meier method, and the survival rates were compared by log-rank test. The association between clinical parameters and SRE was assessed by the Cox proportional hazards regression model, both in bivariate and in multivariable models. Hazard ratios (HRs) are presented with 95% confidence intervals (CI). A preplanned multiple-event analysis was performed using the Andersen-Gill approach, and the robust estimate of variance was used to calculate *P* values [18]. All statistical tests were two-tailed, and *P* < 0.05 was considered significant. Statistical analyses were performed by IBM SPSS 25.0 (SPSS, Inc., Chicago, United States).

Follow-up started at the initiation of ZA treatment and ended at the time of death or last follow-up.

## 3. Results

### 3.1. Clinical Characteristics of Patients

A total of 566 cases were eligible for the efficacy analysis, and the mean follow-up time was 37.2 months (6.6–129.3 months). Patients were divided into two groups: group A (ZA 6–24 months, *n* = 293) and group B (ZA > 24 months, *n* = 273). The median age at bone metastasis was 47 years in both groups. The median time from breast cancer (BC) to bone metastasis (BM) was 30.6 months (0–343.2 months) in group A and 33.9 months (0–279.6 months) in group B (*P* = 0.186). There was a higher proportion of hormone receptor (HR) positive patients and a lower percentage of HER2 positive patients in our study population. Patients were positive for estrogen receptor (ER) in 78.4% of cases (72.0% in group A and 85.3% in group B, *P* < 0.001). The trend was similar for cases with positive progesterone receptor (PR) (65.5% in group A versus 81.0% in group B, *P* < 0.001). Furthermore, 38.9% of cases in group A and 27.1% of cases in group B were HER2 positive (*P* = 0.011). The use of endocrine therapy in these cases was unbalanced (66.2% in group A versus 75.5% in group B, *P* = 0.016), while anti-HER2 target therapy was comparable between groups (19.8% in both groups, *P* = 0.996). There was no significant difference in menstrual status, performance status, pathological subtype, tumor size, lymph node involvement, number of bones involved, stage of bone metastasis, other organ involvement, and chemotherapy between two groups (Table 1).

The duration of ZA treatment was different between the two groups (*P* < 0.001); the median period was 13.9 months in group A (6.0–24.0 months) and 39.3 months in group B (24.2–127.5 months). In 220 cases (80.6%), the infusion interval of ZA was switched to every 2 to 3 months beyond 24 months.

### 3.2. AEs of Long-Term ZA in Breast Cancer Patients

ZA had acute adverse effects, including flu-like symptoms and hypocalcemia, as well as long-term side effects, including renal function impairment, hearing impairment, and ONJ [3–6, 19]. A total of 566 cases were enrolled and were stratified as above. In the current study, we focused on long-term AEs: fifteen cases (2.7%) developed ONJ; nine (3.1%) in group A and six (2.2%) in group B (*P* = 0.606) (Table 2). The median time from ZA initiation to ONJ diagnosis was 22.8 months (5–60 months), and the median age at ONJ occurrence was 53 years (38–73 years). The most common characteristic of these ONJ cases was poor oral hygiene and dental extraction without sufficient intermission to ZA cessation. All cases stopped ZA treatment permanently after ONJ; two patients received surgery to eliminate the osteonecrosis and recovered the jaw function completely.

Five patients (0.9%) were complicated with renal function impairment, two (0.7%) in group A versus 4 (1.1%) in group B (*P* = 0.676) (Table 2). One of these patients was diagnosed with acute renal impairment, but renal function returned to normal after discontinuing ZA. One patient had hearing impairment after 23 months of ZA treatment in group B (0.4%, *P* = 0.482) and recovered her hearing after ceasing ZA.

### 3.3. Distribution of SRES in Breast Cancer with Bone Metastasis

**Figure 1:**
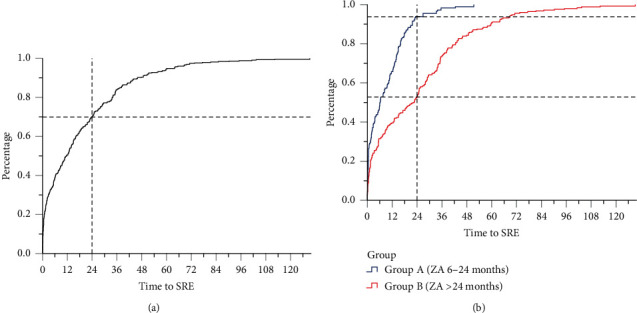
(a) The cumulative incidence of all 426 SREs that occurred after the initiation of ZA in the 241 breast cancer patients with bone metastasis; 70.2% of SREs occurred in the first 24 months. (b) The cumulative incidence of 178 and 248 SREs in group A and group B, respectively. In the first 24 months, 93.8% and 53.2% of SREs occurred in group A (ZA 6–24 months) and group B (ZA > 24 months), respectively.

By the last follow-up, 241 cases (42.6%) developed SREs after ZA treatment; 103 cases in group A (35.2%) and 138 cases in group B (50.6%, *P* < 0.001) (Table 3). Of whom, 127 cases (22.4%) had a single SRE and 114 cases (20.1%) had multiple SREs (*P* = 0.017) (Table 4). Multiple SREs were asynchronous and strictly defined according to a previous report [18, 20]. There were 426 SREs at the last follow-up (Table 5), and the distribution of all SREs was investigated (Figure 1(a)). In all cases, more than 70% of SREs occurred within 24 months of ZA treatment. The peak period of SREs was 24 months in group A, while it extended to 48 months in group B (Figure 1(b)), which implied that the duration of ZA should be individual according to the risk assessment of SRE in metastatic BC.

**Figure 2:**
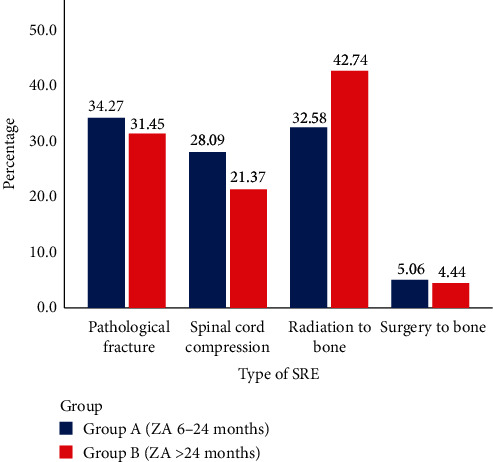
Differences in the types of SREs in breast cancer with bone metastasis between group A (ZA 6–24 months) and group B (ZA > 24 months). The chi-square test revealed that there was no significant difference in the proportion of each SRE type between the two groups (*P* = 0.168).

We then analyzed the frequency of SREs. Although the frequency of various SREs was different, no significant difference was observed between the two groups (*P* = 0.168) (Table 5 and Figure 2). The most common SRE was pathological fracture (61 events, 34.3%), followed by radiation to bone (58 events, 32.6%) in group A. Radiation to the bone was the most frequent (106 events, 42.7%), followed by pathological fracture (78 events, 31.2%) in group B. Spinal cord compression was the third SRE in both groups (28.1% in group A versus 21.4% in group B). Surgery to bone was similarly low in both groups (5.1% in group A versus 4.4% in group B). This suggested that long-term use of ZA had no significant impact on the incidence of SRE type.

### 3.4. Efficacy of ZA on SREs in Breast Cancer with Bone Metastasis

**Figure 3:**
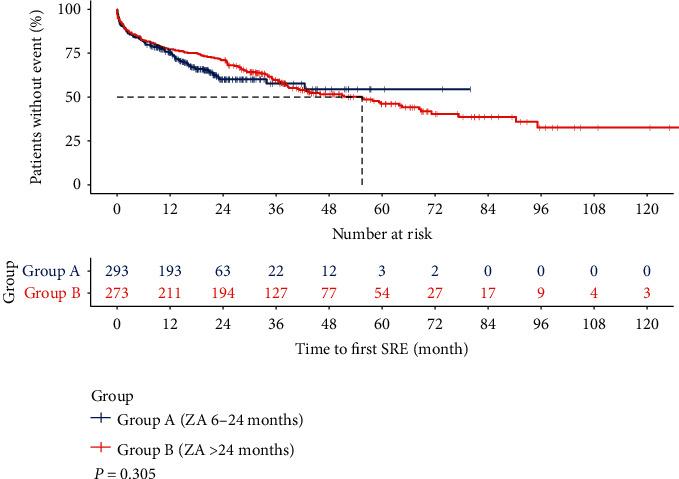
Difference in skeletal-related event-free survival in breast cancer with bone metastasis between group A (ZA 6–24 months) and group B (ZA > 24 months). Kaplan–Meier analysis was used to compare the SRE free survival between the two groups.

Of all 426 SREs, 178 were in group A and 248 in group B (Tables 4 and 5, *P* = 0.017). The mean annual incidence rate of SREs was 0.3 (0–3.1) events in group A and 0.2 (0–1.0) events (*P* = 0.269), which indicated that long-term ZA treatment reduced the cumulative incidence of SREs, although the change was not significantly different. The median survival time to first SRE was not reached in group A and took 55.5 months in group B (0.1–129.3 months), but, again, the difference was not obvious (*P* = 0.305) (Figure 3). These results suggested that extension of ZA treatment beyond 24 months might not decrease the cumulative incidence risk of SRE.

**Figure 4:**
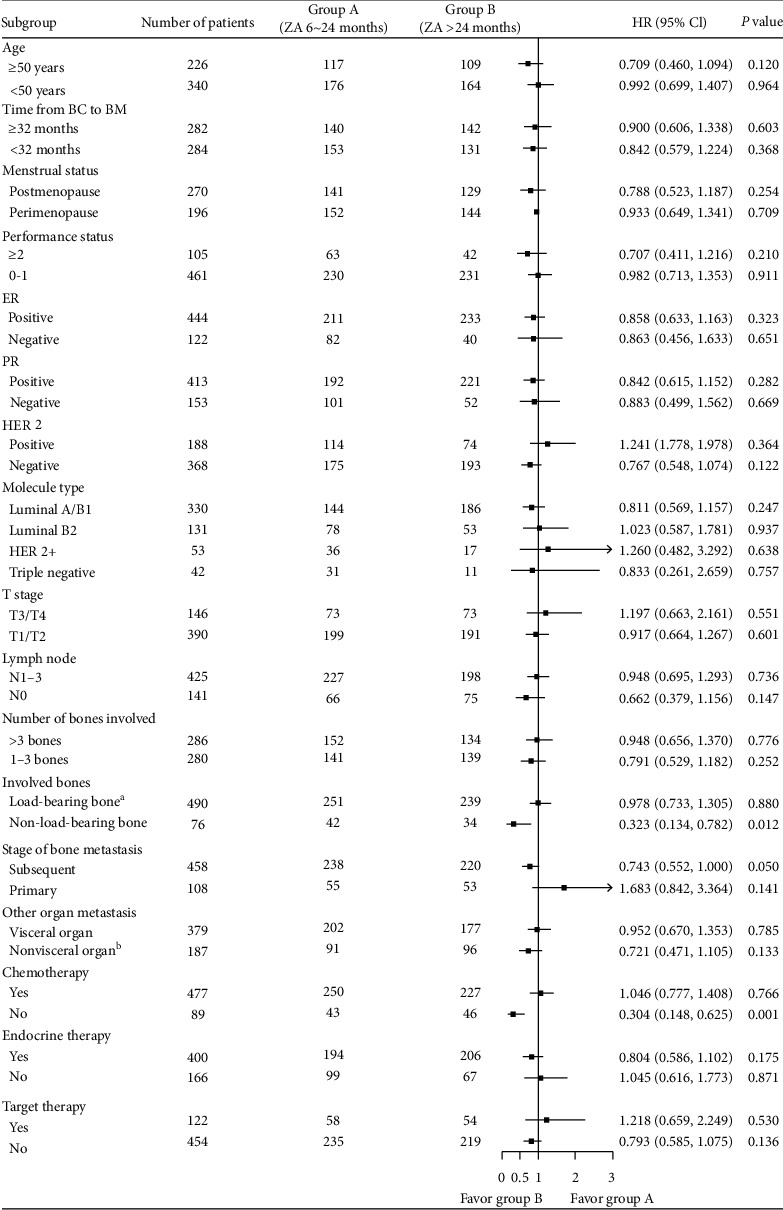
Forest plot depicting the hazard ratios of different subgroups in group A (ZA 6–24 months) and group B (ZA > 24 months). The square data point represents the hazard ratio, and the endpoints represent 95% confidence intervals. The hazard ratio and *P* value were calculated by the Cox regression test. a: Load-bearing bones consist of the spine, pelvis, and the limbs; these are strongly influenced by muscle strength, which affects the healing of fractures, bone grafts, osteotomies, and arthrodesis. The remainder are non-load-bearing bones, such as the skull, rib, clavicle, humerus, and ulna. b: Nonvisceral organ metastasis includes bone, lymph node, soft tissue, chest wall, and ipsilateral breast cancer.

Next, we performed subgroup analysis to explore who could benefit from long-term treatment. It was suggested that cases with involvement of non-load-bearing bones (HR, 0.323; 95% CI: 0.134–0.782; *P* = 0.012), and those who received systematic anticancer therapy without chemotherapy (HR, 0.304; 95% CI: 0.148–0.625, *P* = 0.001) might benefit from long-term ZA treatment (Figure 4). Load-bearing bone consists of the spine, pelvis, and the limbs, which are strongly influenced by muscle strength, and affects the healing of fractures, bone grafts, osteotomies, and arthrodesis. The remainder are non-load-bearing bones, such as the skull, rib, clavicle, humerus, and ulna [21].

### 3.5. Factors Affecting the Risk of SREs after ZA Treatment

Cox proportional hazards regression analysis was applied in bivariate and multivariable models in order to further investigate factors that predict the risk of SREs. Cofactors included age at BM, performance status, menstrual status, tumor size, lymph node involvement, ER, PR, HER2 expression, molecular subtype, number of bones involved, site of the involved bone, stage of BM, other organ metastases, time from BC diagnosis to BM, and the duration of ZA treatment (Table 6). Bivariate model analysis suggested that poor performance status (HR, 2.054; 95% CI, 1.534–2.750; *P* = 0.001), high number of involved bones (HR, 1.309; 95% CI, 1.014–1.689; *P* = 0.038), and nonvisceral organ metastasis (HR, 1.407; 95% CI, 1.087–1.820; *P* = 0.009) increased the risk of SRE. Nonvisceral organ metastasis includes bone, lymph node, soft tissue, chest wall, and ipsilateral breast cancer [22]. In multivariate Cox regression analysis, we included these three factors and factors that clearly increased the risk of SREs according to previous studies [23, 24]: the site of the involved bone and the time from BC to BM. It was shown that patients with performance status and nonvisceral organ metastasis are at higher risk of SREs.

## 4. Discussion

This was the first retrospective, self-control study to compare long-term ZA (>24 months) to short-term ZA (≤24 months) in the treatment of BC patients with BM from southern China. Our findings were different to those of previous reports [3–7], in which ZA was compared with either placebo or another bisphosphonate [10], and the observed time was within 25 months [3, 5, 6, 8, 25]. This study aimed to determine whether longer ZA treatment was as safe as conventional treatment on the cumulative incident risk of AEs and whether longer ZA beyond 24 months was superior to short-term treatment in real-world observation.

Long- and short-term ZA treatment was similarly safe to that of short-term ZA for ABC with BM, which did not increase the risk of ONJ, renal function impairment, or hearing impairment from our analysis (Table 2). Bisphosphonates were generally well tolerated, with a low incidence renal dysfunction and ONJ [26, 27]. BPs significantly increased the occurrence of ONJ in patients exposed to bisphosphonates, and the risk of ONJ went up with the cumulative time and dose of ZA exposure [28]. BPs were firstly reported to be well tolerated beyond 24 months in a small number of patients [15]. In patients who developed ONJ in this study, the median exposure time to ZA was 22.8 months (5–60 months) which indicated that extension of ZA did not increase the risk of ONJ with regard to occurrence time and incidence risk. The common characteristic in these ONJ cases was poor oral hygiene and tooth extraction without a sufficient withdrawal window of ZA, which may have led to ONJ. The Updated ASCO Committee Consensus suggests that initiation of BMA therapy should be delayed for 14 to 21 days to allow for wound healing [9]. All patients should be advised to undergo regular oral examination and maintain good oral hygiene.

More than 70% of SREs occurred during the first 24 months in all cases. The peak period of SREs was within 24 months in group A and extended to 48 months in group B. This finding may explain why the ASCO could not recommend the optimal duration of BP therapy and that this should be individual according to the patient's condition [9, 11]. The frequency of SRE types was not obviously diverse between the groups (Figure 2), but radiation to bone was the most common SRE in long-term groups. The skeletal complications often require clinical management, including radiation or surgery [29]. The risk of SREs and the need to control bone lesions increased throughout the trajectory of metastatic breast cancer [9], which may explain the proportion of radiation to the bone raised in a long-term group (Figure 2).

Extension of ZA treatment beyond 24 months might not reduce the annual incidence of SREs compared to the duration of conventional treatment. Most cases (80.6%) switched the delivery interval to every 2–3 months after 24 months. It was reported that decreasing administration of ZA to a 12-weekly regimen after 12–15 months of monthly treatment would not affect the skeletal morbidity [10]. Thus, the interval change in our study might not influence the real-world result. The cumulative SRE risk in our report was similar to that reported in previous prospective clinical trials [10, 30], which indicated that our results were both objective and factual. In our clinical experience, some cases could really benefit from long-term treatment.

We then aimed to determine who can benefit from long-term ZA treatment. Subgroup analysis suggested that patients with non-load-bearing bone involvement or those who did not receive chemotherapy might profit from long-term therapy (Figure 4).

Multivariate analysis found that poor performance status and nonvisceral organ metastasis predicted a higher risk of SRE (Table 6). Poor performance might be reflected by pathological fracture or spinal cord compression. Furthermore, advanced breast cancer with bone-only involvement had better prognosis and longer survival time, in which the risk of SRE would increase as survival time [31]. However, patients with bone metastases would suffer from numerous SREs, and the risk of subsequent SREs clearly increased once SRE started compared to before treatment [32, 33].

To date, no prospective clinical study provided evidence that bone-modifying agents should be continued or stopped at a defined time. Since the risk of SREs existed, the expert panel recommended continuation of therapy beyond 2 years but always based on an individual risk assessment [9, 11, 21]. Our study provided clinical experience that the extension of ZA beyond 24 months was safe but might not significantly decrease the risk of skeletal morbidity, compared to ZA within 24 months. However, since this is a retrospective study, the data might be biased, especially for multiple SERs and complex SREs. Therefore, the clinical benefit of long-term ZOL in BC patients with BM remains to be answered in additional prospective trials.

## Figures and Tables

**Table 1:** Clinical characteristics of patients with advanced breast cancer and bone metastasis between group A (ZA 6–24 months) and group B (ZA > 24 months).

Parameter		Group A (ZA 6–24 months, *n* = 293)	Group B (ZA > 24 months, *n* = 273)	*P* value
Age at BM	Median (range)	47 (19, 77)	47 (25, 76)	0.869^*∗*^
Time from BC to BM	Median (range)	30.6 (0, 343.2)	33.9 (0, 279.6)	0.186^*∗*^
Age group	<50 years	176 (60.1%)	164 (60.1%)	0.999
	≥50 years	117 (39.9%)	109 (39.9%)	
Menstrual status^a^	Perimenopause	152 (51.9%)	144 (52.7%)	0.836
	Postmenopause	141 (48.1%)	129 (47.3%)	
Performance status	0–1	230 (78.5%)	231 (84.6%)	0.061
	≥2	63 (21.5%)	42 (15.4%)	
Pathological subtype	Invasive ductal carcinoma	276 (94.2%)	261 (95.6%)	0.707
	Invasive lobular carcinoma	11 (3.8%)	7 (2.6%)	
	Other type	6 (2.0%)	5 (1.8%)	
T stage	T1/T2	199 (67.9%)	191 (70.0%)	0.119
	T3/T4	73 (24.9%)	73 (26.7%)	
	Unknown	21 (7.2%)	9 (3.3%)	
Lymph node	N0	66 (22.5%)	75 (27.5%)	0.174
	N1–3	227 (77.5%)	198 (72.5%)	
ER	Negative	82 (28.0%)	40 (14.7%)	<0.001
	Positive	211 (72.0%)	233 (85.3%)	
PR	Negative	101 (34.5%)	52 (19.0%)	<0.001
	Positive	192 (65.5%)	221 (81.0%)	
HER2	Negative	175 (59.7%)	193 (70.7%)	0.011
	Positive	114 (38.9%)	74 (27.1%)	
	Unknown	4 (1.4%)	6 (2.2%)	
Number of bones involved	≤3	141 (48.1%)	139 (50.9%)	0.507
	>3	152 (51.9%)	134 (49.1%)	
Involved bones^b^	Non-load-bearing bone	42 (14.3%)	34 (12.5%)	0.512
	Load-bearing bone	251 (85.7%)	239 (87.5%)	
Stage of bone metastasis	Primary	55 (18.8%)	53 (19.4%)	0.846
	Subsequent	238 (81.2%)	220 (80.6%)	
Other organ metastasis^c^	Nonvisceral organ	91 (31.0%)	96 (35.2%)	0.299
	Visceral organ	202 (69.0%)	177 (64.8%)	
Systematic therapy	Chemotherapy	250 (85.3%)	227 (83.2%)	0.478
	Endocrine therapy	194 (66.2%)	206 (75.5%)	0.016
	Target therapy	58 (19.8%)	54 (19.8%)	0.996

BC: breast cancer, BM: bone metastasis, M: month, ER: estrogen receptor, PR: progesterone receptor; ZOL: zoledronic acid, CNS: central nervous system. ^*∗*^Data are presented as median (range) and are compared using the Mann–Whitney *U* Test. ^a^Perimenopause is defined as bone metastasis that occurs before menopause, while bone metastasis that occurs after menopause is defined as postmenopause. ^b^Load-bearing bones consist of the spine, pelvis, and the limbs; these are strongly influenced by muscle strength, which affects the healing of fractures, bone grafts, osteotomies, and arthrodesis. The remainder are non-load-bearing bones, such as the skull, rib, clavicle, humerus, and ulna. ^c^Nonvisceral organ metastasis includes bone, lymph node, soft tissue, chest wall, and ipsilateral breast cancer. ^d^Cases were grouped by bone metastasis occurring at, or after, the diagnosis of breast cancer. Data are presented as number (percentage) of patients unless otherwise indicated. All categorical variables were compared by the Pearson *χ*2 or Fisher's exact test.

**Table 2:** Long-term adverse effects of ZA in advanced breast cancer with bone metastasis^*∗*^.

Parameter		Group A (ZA 6–24 months) *n* = 293	Group B (ZA > 24 months) *n* = 273	*P* value^*∗∗*^
ONJ	No	284 (96.9%)	267 (97.8%)	0.606
	Yes	9 (3.1%)	6 (2.2%)	
Renal impairment	No	291 (99.3%)	270 (98.9%)	0.676
	Yes	2 (0.7%)	3 (1.1%)	
Hearing impairment	No	293 (100.0%)	273 (99.7%)	0.482
	Yes	0 (0.0%)	1 (0.4%)	

ONJ: osteonecrosis of jaws; AE: adverse effect. ^*∗*^In the safety analysis, the enrolled cases included cases without SRE before the initiation of ZA, mentioned previously, and cases with SRE before the initiation of ZA. All were compared by Fisher's exact test.

**Table 3:** Number of cases with SREs in ABC with bone metastasis according to the initiation of ZA^*∗*^.

SRE	Group A (ZA 6–24 months) *n* = 293	Group B (ZA > 24 months) *n* = 273	*P* value
No	190 (64.8%)	135 (49.4%)	<0.001
Yes	103 (35.2%)	138 (50.6%)	

SRE: skeletal-related event, ABC: advanced breast cancer, and ZA: zoledronic acid. ^*∗*^All cases without SRE before the initiation of ZA were divided into subgroups by the occurrence of SRE after the initiation of ZA. The difference between subgroups was compared by the Pearson chi-square test.

**Table 4:** Quantity of SREs in ABC with bone metastasis after treatment with ZA.

	Group A (ZA 6–24 months) *n* = 293	Group B (ZA > 24 months) *n* = 273	*P* value
No events^a^	190 (64.8%)	135 (49.4%)	0.017
Single event^b^	58 (19.8%)	69 (25.3%)	
Multiple events^c^	45 (15.4%)	69 (25.3%)	

SRE: skeletal-related event, MBC: metastatic breast cancer, and ZA: zoledronic acid. ^a^No event was defined as no SRE after the initiation of ZA. ^b^Single event indicated that the enrolled cases had one single SRE after the initiation of ZA. ^c^Multiple events indicated that the enrolled cases had two or more SREs after treatment with ZA.

**Table 5:** SREs in ABC with bone metastasis after treatment with ZA^*∗*^.

Type of SRE	Group A (ZOL 6–24 months) *E* = 178^a^	Group B (ZOL > 24 months) *E* = 248^b^	*P* value
Pathological fracture	61 (34.3%)	78 (31.2%)	0.168
Spinal cord compression	50 (28.1%)	53 (21.4%)	
Radiation to bone	58 (32.6%)	106 (42.7%)	
Surgery to bone	9 (5.1%)	11 (4.4%)	

E: event, SRE: skeletal-related event, ABC: advanced breast cancer, and ZA: zoledronic acid. ^*∗*^All SREs are discussed separately and were compared by Pearson *χ*^2^ test. ^a, b^A total of 178 SREs occurred in all patients in group A and 250 SREs in group B, including the first and subsequent SREs.

**Table 6:** Cox regression analysis of breast cancer with bone metastasis following ZA treatment.

Parameter		Univariate			Multivariate		
		HR	95% CI	*P* value	HR	95% CI	*P* value
Age	<50 years						
	≥50 years	0.980	(0.756, 1.270)	0.876			
Menstrual status	Perimenopausal						
	Postmenopausal	0.884	(0.685, 1.141)	0.345			
ER	Negative						
	Positive	1.057	(0.766, 1.459)	0.734			
PR	Negative						
	Positive	1.038	(0.773, 1.393)	0.805			
HER2	Negative						
	Positive	1.106	(0.869, 1.408)	0.414			
Molecular type	Luminal A/B1						
	Luminal B2	1.141	(0.832, 1.565)	0.412			
	HER2+	1.019	(0.637, 1.629)	0.939			
	Triple negative	1.055	(0.629, 1.770)	0.839			
Tumor size	T1–T2						
	T3–T4	0.936	(0.692, 1.267)	0.670			
Lymph node	N0						
	N1–N3	1.147	(0.856, 1.539)	0.359			
Performance status	0–1						
	≥2	2.054	(1.534, 2.750)	<0.001	2.073	(1.539, 2.793)	<0.001^*∗*^
No. of involved bones	1–3 bones						
	>3 bones	1.309	(1.014, 1.689)	0.038	1.209	(0.913, 1.603)	0.186
Involved bones	Non-load-bearing bone						
	Load-bearing bone	1.305	(0.879, 1.937)	0.187	1.111	(0.722, 1.711)	0.632
Stage of bone metastases	Primary						
	Subsequent	1.159	(0.822, 1.633)	0.400			
Other organ metastasis	Visceral organ						
	Nonvisceral organ	1.407	(1.087, 1.820)	0.009	1.513	(1.163, 1.968)	0.002^*∗*^
Time from BC to BM group	<32 months						
	≥32 months	0.832	(0.645, 1.072)	0.154	0.971	(0.726, 1.298)	0.842
ZOL group	∼24 months						
	>24 months	0.868	(0.662, 1.138)	0.305			

ER: estrogen receptor, PR: progesterone receptor, ZA: zoledronic acid, and HR: hazard ratio. ^*∗*^Backward stepwise (conditional LR) was selected for multivariate Cox regression analysis.

## Data Availability

The patient data used to support the findings of this study are available from the corresponding author upon request.
